# Bispecific Antibodies Versus Chimeric Antigen Receptor T‐Cell Therapy in Relapsed/Refractory Diffuse Large B‐Cell Lymphoma: A Comparative Narrative Review of Efficacy, Safety, and Accessibility

**DOI:** 10.1002/cam4.71562

**Published:** 2026-01-29

**Authors:** Dana Sofian Abou, Husna Irfan Thalib, Fayza Akil, Samia Zuhair Sabbagh, Hala Sofian Abou, Mable Pereira, Fatma ElSayed Hassan

**Affiliations:** ^1^ Pharmacy Program Batterjee Medical College Jeddah Saudi Arabia; ^2^ General Medicine Practice Program Batterjee Medical College Jeddah Saudi Arabia; ^3^ School of Medicine Lincoln American University Georgetown Guyana; ^4^ General Medicine Practice Program, Department of Physiology Batterjee Medical College Jeddah Saudi Arabia

**Keywords:** accessibility, bispecific antibodies, CAR T cell therapy, efficacy, relapsed/refractory diffuse large B‐cell lymphoma, safety

## Abstract

**Introduction:**

Diffuse large B‐cell lymphoma (DLBCL) is the most common subtype of non‐Hodgkin lymphoma, and despite advances in frontline therapies such as rituximab, cyclophosphamide, doxorubicin hydrochloride (hydroxydaunorubicin), vincristine sulfate (Oncovin), and prednisone, approximately 30%–40% of patients develop relapsed or refractory (rel/ref) disease. This subgroup has historically faced poor prognoses with limited treatment options, prompting the development of novel immunotherapeutic strategies. Chimeric antigen receptor T‐cell (CAR T) therapy and bispecific antibodies (BsAbs) have emerged as transformative approaches in this setting.

**Methods:**

This narrative review compares these therapies across multiple domains, including mechanisms of action, clinical efficacy, safety profiles, logistics, cost, and accessibility.

**Results:**

CAR T therapies have demonstrated durable complete response rates (40%–60%) and extended progression‐free survival (median 11–12.5 months), but they are limited by complex manufacturing, high cost, and potentially severe toxicities. In contrast, BsAbs offer immediate, off‐the‐shelf availability, with promising efficacy and a more favorable safety profile that enables outpatient administration, although long‐term durability remains under investigation.

**Conclusion:**

This review provides clinicians with a comprehensive comparison to support evidence‐based treatment selection in rel/ref DLBCL.

**Abbreviations**
BsAbs: bispecific antibodies
CAR T: chimeric antigen receptor T‐cell
CR: complete response
CRS: cytokine release syndrome
ctDNA: circulating tumor DNA
DLBCL: diffuse large B‐cell lymphoma
EAP: expanded access program
EMA: European Medicines Agency
FDA: U.S. Food and Drug Administration
GMP: good manufacturing practice
ICANS: immune effector cell‐associated neurotoxicity syndrome
IPI: international prognostic index
LDH: lactate dehydrogenase
MAIC: matching‐adjusted indirect comparison
ORR: overall response rate
OS: overall survival
PET: positron emission tomography
PFS: progression‐free survival
QALY: quality‐adjusted life year
R/R: relapsed/refractory
SUVmax: maximum standardized uptake value
TRUCK: T‐cells redirected for universal cytokine‐mediated killing

## 1 Introduction

Diffuse Large B‐cell Lymphoma (DLBCL), the most prevalent variant of non‐Hodgkin's lymphoma (NHL), is caused due to the malignant transformation of B lymphocytes. A very aggressive form of cancer, it has become a serious as well as an escalating public health issue, with expected increases reaching 60,000 newly diagnosed cases in the combined United States of America and Western Europe in 2023. The expected rise in cases would continue due to the expected demographic shift towards an older population, surpassing 32,000 newly diagnosed cases only in the United States in 2025 [1]. Despite the available progressions in initial therapy, namely the introduction of the monoclonal antibody rituximab, which binds specifically to the cluster of differentiation (CD) 20 antigen on B cells, within conventional cyclophosphamide, doxorubicin, vincristine, and prednisone (CHOP)‐based therapeutic regimens, the survival rates have improved only from 57% to 70% overall [2]. Nevertheless, about 30%–40% of newly diagnosed cases suffer from relapsed/refractory diseases. The condition of being relapsed/refractory poses particularly difficult therapeutic challenges, as well as a high possibility of mortality [3, 4].

The treatment scenario for rel/ref DLBCL has recently been revolutionized by some newer immunotherapy innovations. Chimeric antigen receptor T‐cell therapy is a personalized therapeutic approach in which a patient's own T cells are collected, engineered to express receptors recognizing CD19 on tumor cells, and then administered for targeted therapeutic action. The results have been phenomenal, yielding long‐lasting responses even within highly pre‐treated, refractory, and relatively untreatable patients [5, 6]. There have been serious challenges in formally placing CAR‐T cell therapy on a larger scale, including prolonged processing phases, high costs, limited accessibility at specialized centers, and high toxicity, encompassing cytokine releases, neurological, and other serious toxicities [5]. On the contrary, bispecific antibodies, as accessible ‘off‐the‐shelf’ biologics, offer immediate alternatives as future therapeutic avenues, simultaneously interacting with both T cells as well as malignant B cells, recognizing CD3 on T cells and CD20/CD19 antigens on B cells, leading to targeted killing. The recently approved glofitamab and epcoritamab have recently exemplified encouraging responses in rel/ref DLBCL, retaining relatively safe therapeutic indexes, as well as relatively easy accessibility within large‐scale practice [7, 8]. Nevertheless, long‐term follow‐through on durability as well as curability within these novel therapeutic prospectives remains pending.

Taking into consideration the unique features, benefits, as well as challenges associated with these two revolutionary immunotherapy regimes, it is paramount to conduct a systematic comparison in order to make informed decisions. This review will attempt to encompass the mechanisms of action, approval status for use, efficacy results, rates, pharmacovigilance, accessibility, cost, patient selection, mechanisms of resistance, novel combinations, quality of life, as well as implementation in relation to a majority of healthcare systems. It is hoped that, through our review, evidence will be provided to inform health practitioners on how to cope within the emerging therapeutic approach available for rel/ref DLBCL.

## 2 Mechanism of Action

CAR‐T cell therapy, as well as BsAbs, is truly novel therapeutic modalities available for the management of rel/ref DLBCL. Although both have the capability of using T‐cell cytotoxicity against malignant B cells, their engineering design principles are entirely different, thereby possessing distinct properties.

### 2.1 CAR T‐Cell Therapy: Precision Cellular Reprogramming Through Synthetic Biology

**FIGURE 1:**
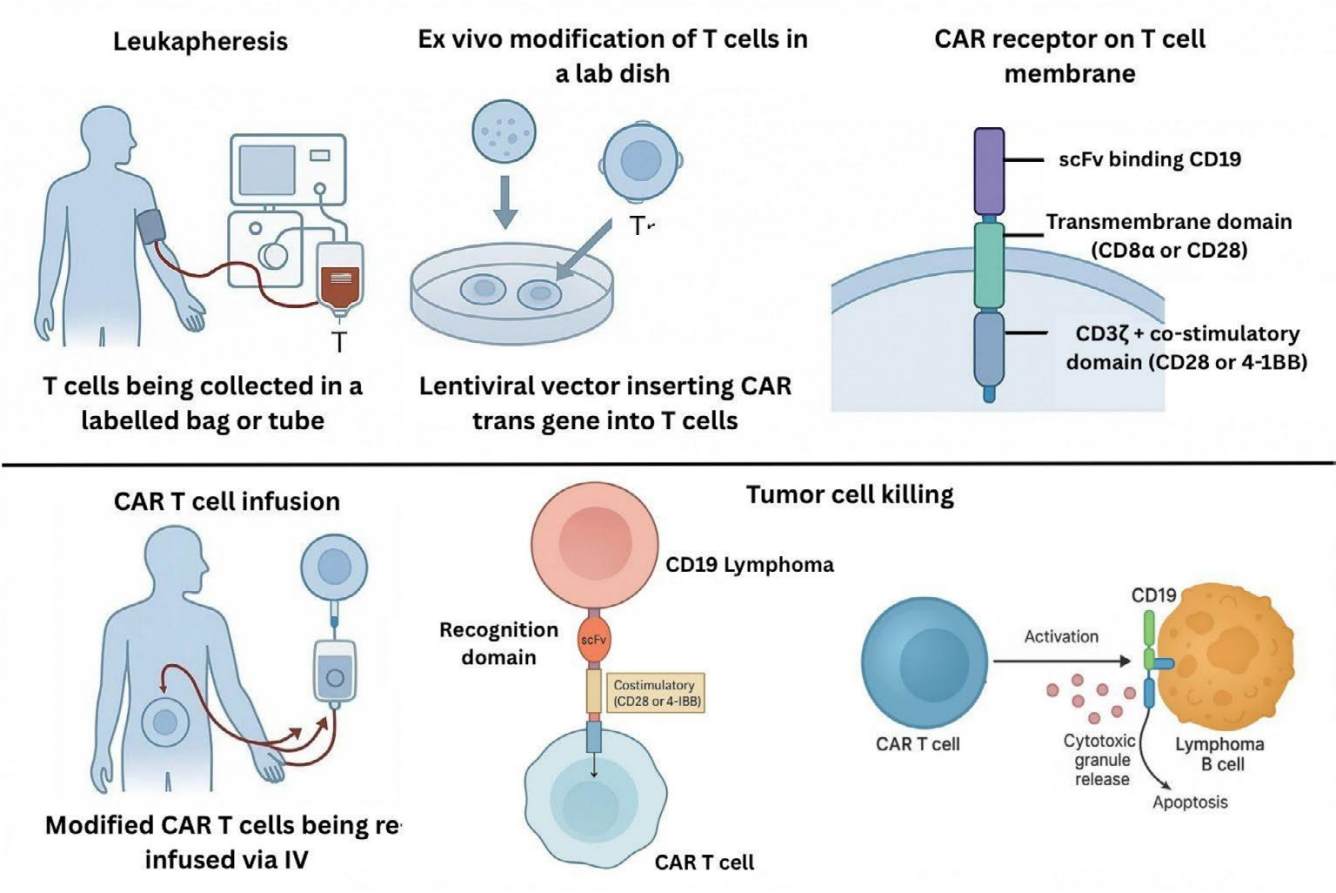
Mechanism of CAR T‐cell therapy in diffuse large B‐cell lymphoma. CAR, chimeric antigen receptor; CRS, cytokine release syndrome; DLBCL, diffuse large B‐cell lymphoma; ICANS, immune effector cell‐associated neurotoxicity syndrome; scFv, single‐chain variable fragment.

The CAR T‐cell therapy represents a quantum leap within personalized cellular engineering. The complex procedure involves, following a first step of leucapheresis for the isolation of autologous T cells, a sophisticated genetic ex vivo manipulation based on the use of viral vectors, mainly lentiviral systems, to knock‐in CARs conferring nanomolar affinity for CD19 antigens. Contemporary CARs have a trimodular configuration, consisting of an extracellular recognition moiety, usually a humanized single‐chain variable fragment, scFv, which enables MHC‐independent CD19 specificity to circumvent antigen presentation escape mechanisms [9, 10]. This is logically underpinned by a membrane‐anchoring domain, usually derived from CD8 α/CD28, which provides optimal membrane localization, as well as activated by intracellular signaling pathways, such as CD3ζ signaling for initial cell activation, along with costimulatory domains, CD28, 4‐1BB, CD137. The choice of costimulation signaling plays a crucial role, as CD28 signaling supports maximal proliferation, while 4‐1BB signaling supports long‐term persistence, thereby significantly reducing exhaustion of CAR T cells [9, 11]. The structural arrangement and activation signaling of a CAR T‐cell recognizing CD19^+^ lymphoma cells are depicted in Figure 1.

After lymphodeletion and cell reinjection, a striking degree of clonal expansion of CAR T cells has been observed, often 1000 to 10,000‐fold from days 7–14 following cell infusions [12]. In response to CD19^+^ lymphoma cells, they form ordered immune synapses and execute directed cytotoxicity via pore‐mediated killing utilizing perforins as well as apoptotic pathways initiated by granzymes [13]. Because of this remarkable specificity, bolstered by the action of local cytokine gradients, rates of complete response have reached 40%–54% in extensively pretreated patients [9, 10].

Nevertheless, a number of challenges remain. The production procedure is intricate, as it takes 2–4 weeks ex vivo, thereby creating a susceptible window for progression of diseases. High‐grade toxicities primarily related to cytokine release syndrome (CRS) and immune effector cell‐associated neurotoxicity syndrome (ICANS) require intensive care, which is usually taken up in specialized centers [14]. In addition, antigen escape, specifically CD19 loss, has driven the development of the next‐generation dual‐targeted “armored” CARs, which can secrete immunomodulatory cytokines to modify the tumor microenvironment [15].

### 2.2 Bispecific Antibodies: Molecular Orchestrators of Immune Synapse Formation

**FIGURE 2:**
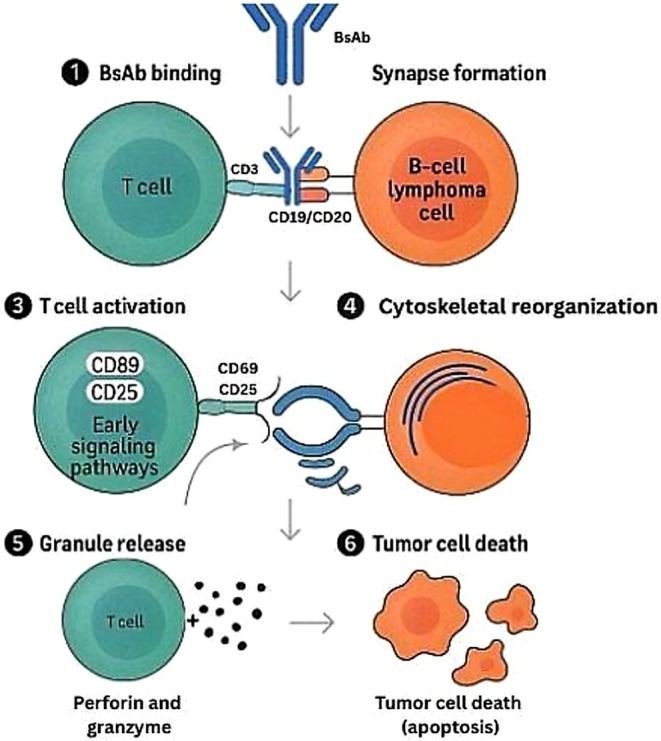
Mechanism of bispecific antibody therapy in diffuse large B‐cell lymphoma. Bispecific antibodies (BsAbs) simultaneously bind CD3ε on cytotoxic T cells and CD19/CD20 on malignant B cells, physically bridging the two cells and forming an immune synapse that triggers targeted T‐cell–mediated lysis of lymphoma cells. BsAb, bispecific Antibody; CD3, cluster of differentiation 3; CD19, cluster of differentiation 19; CRS, cytokine release syndrome; DLBCL, diffuse large B‐Cell lymphoma; ICANS, immune effector cell‐associated; TCR, T‐cell receptor.

BsAbs represent a straightforward, off‐the‐shelf option sidestepping the requirement for cell modulation. These molecules are engineered to bind CD3ε on T cells as well as CD19/CD20 on cancerous B cells, thereby instructing the patient's own T cells to target lymphoma cells [8]. BsAbs can be developed as immunoglobulin G (IgG)‐based constructs, whose Fc domains can be utilized for the recycling of neonatal fragment crystallizable receptors, adding to their long half‐life, as well as potentially facilitating immune cell interaction as a result of Fc gamma receptor binding [16]. Others include fragment‐based BiTE, DART, and TandAb, demonstrating improved tissue distribution, although having shorter half‐lives, often mandating continuous, stepwise infusions [13, 17]. The dual‐component approach taken by bispecifics, interacting with both T cells and cancerous cells, is depicted in Figure 2.

Their pharmacodynamics are based on affinity for CD3 binding sites, a phenomenon which has to be precisely optimized to achieve a balance between strong T‐cell activation and the possibility of cytokine toxicity. Novel administration schedules, especially step‐up schedules, have demonstrated efficacy in reducing CRS in early‐stage therapeutic strategies [18]. After administration, BsAbs cause T‐cell activation, a process that involves the formation of strategic immunological synapses, identified as a phenomenon of supramolecular assembly cluster formation, accompanied by a temporal expression of markers such as CD69, CD25, and CD137, along with cytoskeletal rearrangement, allowing directional delivery of cytotoxic granules to the tumor cells [16, 19, 20].

In contrast to CAR T cells, the immune effects of BsAbs are transient, following the levels of the drug in the blood, making it possible to have a controllable toxicity profile. Hence, fewer occurrences of grade ≥ 3 CRS or neurotoxicity have been observed [21]. Nevertheless, challenges for BsAbs also include optimizing affinity ratios to limit toxicities, dealing with immune suppression in the tumor microenvironment, as well as outpatient administration regimens to maintain T‐cell function [13, 22].

Recent advances have enabled a further convergence of CAR‐T cell therapy and BsAbs. For instance, fourth‐generation CARs, also referred to as T‐cells redirected for universal cytokine‐mediated killing (TRUCKs), have been designed to secrete interleukin (IL)‐12 only in response to tumor cells, thereby allowing microenvironmental conditioning without unleashing systemic levels of cytokine [15]. Meanwhile, within the BsAbs framework, affinity‐modulated constructs, exemplified by the investigational bispecific antibody drug TNB‐486, have reduced CD3 binding affinity to decrease cytokine release while maintaining anti‐tumor function, a step towards completely outpatient‐ready therapy [23].

Based on Table 1, it can be noted that the comparison between CAR T cell therapy and BsAbs becomes apparent. The CAR T cell therapy, being a personalized, in‐patient intensive approach, provides enduring immune surveillance, but the approach poses a significant toxicity burden. The advantage of BsAbs includes immediate use, outpatient accessibility, as well as a more acceptable toxicity profile, though it encompasses a transient response duration as well as emerging mechanisms for overcoming the tumor microenvironment‐mediated inhibition [13, 16, 22].

**TABLE 1:** Key differences between CAR T cell therapy and BsAbs in rel/ref DLBCL [7, 8, 9, 10, 16, 18].

Feature	CAR T‐cell therapy	BsAbs
Therapy type	Autologous cellular therapy	Off‐the‐shelf recombinant proteins
Target antigen	CD19	CD19 or CD20
T‐cell engagement	Genetically engineered T cells expressing CAR	Simultaneous binding of CD3 on T cells and CD19/CD20 on B cells
Manufacturing time	Several weeks (patient‐specific)	Immediate availability
Administration	Single infusion, inpatient setting	Multiple doses, often outpatient/subcutaneous
T‐cell persistence	Long‐lived memory T cells	Shorter half‐life: persistence depends on dosing
Immune activation	Self‐amplifying in vivo	Dose‐dependent activation
Safety profile	High risk of CRS and neurotoxicity (ICANS)	Generally milder CRS and fewer neurotoxic events
Toxicity management	Requires specialized centers and intensive monitoring	Step‐up dosing mitigates CRS risk
Cost and accessibility	High cost, limited to specialized centers	Lower cost, more accessible
Tumor microenvironment modulation potential	‘Armored’ CARs secrete cytokines (e.g., IL‐12) to modulate microenvironment	Emerging low‐affinity CD3 BsAbs reduce toxicity while maintaining efficacy
Treatment setting	Hospital‐based due to complexity and side effects	Potential for outpatient administration

Abbreviations: BsAbs, bispecific antibodies; CAR T, chimeric antigen receptor T‐cell therapy; CD, cluster of differentiation; CRS, cytokine release syndrome; ICANS, immune effector cell–associated neurotoxicity syndrome; IL‐12, interleukin 12.

Taken together, these therapeutic areas encompass overlapping but distinct and complementary approaches within the immunotherapy toolkit for the treatment of rel/ref DLBCL.

## 3 Approval Timelines and Regulatory Pathways

In the treatment of relapsed/refractory (rel/ref) DLBCL, regulatory approval timelines and pathways differ significantly between CAR T‐cell therapies and bispecific antibodies (BsAbs), as well as across global agencies such as the U.S. Food and Drug Administration (FDA) and the European Medicines Agency (EMA) (Table 2). CAR T therapies have largely been approved in the U.S. through accelerated or expedited pathways, allowing for rapid access based on single‐arm trial data. For example, axicabtagene ciloleucel was approved by the FDA in 2017, followed by tisagenlecleucel in 2018 and lisocabtagene maraleucel in 2021, all supported by pivotal trials such as ZUMA‐1, JULIET, and TRANSCEND, respectively [24, 25, 26, 27, 28, 29, 30]. These approvals often come with post‐marketing requirements, including confirmatory trials. In contrast, the EMA typically utilizes full authorization pathways, with longer review periods; for example, tisagenlecleucel received EMA approval only on June 28, 2018 [10]. Similarly, the BsAb glofitamab received conditional approval in Canada in March 2023, and a positive opinion from EMA in April 2023, but had not yet achieved full European authorization at the time of reporting [27]. The variation in approval timelines between the FDA and EMA largely reflects differences in their regulatory philosophies. The FDA often utilizes accelerated approval pathways for therapies addressing serious or life‐threatening conditions, such as relapsed/refractory DLBCL. This allows treatments like CAR T‐cell therapies to be approved based on early‐phase, single‐arm trials showing strong clinical benefit, with the requirement for post‐marketing confirmatory studies. In contrast, the EMA tends to follow a more conservative and comprehensive approach, requiring more mature or comparative data before granting full marketing authorization. This results in longer review periods but reflects a greater emphasis on long‐term safety and efficacy. Similarly, bispecific antibodies such as glofitamab have seen faster conditional approvals in some regions like the U.S. and Canada, while still awaiting full EMA authorization, further highlighting these regulatory differences [24, 25, 26, 27, 28, 29, 30].

**TABLE 2:** Approval timelines and regulatory type for CAR T vs. BsAbs.

Therapy type	Product name	FDA approval year	EMA status and year	FDA pathway	EMA pathway
CAR T cell therapy	Axicabtagene ciloleucel (Yescarta)	2017	Authorized 2018	Accelerated [24, 25]	Full [26]
	Tisagenlecleucel (Kymriah)	2018	Full authorization—28 June 2018	Accelerated [24]	Full [10]
	Lisocabtagene maraleucel (Breyanzi)	2021	No EMA date found	Accelerated [25]	Not specified
BsAbs	Glofitamab	Under review	Positive opinion—April 2023	Pending/Conditional [27]	Conditional [27]

Abbreviations: BsAbs, bispecific antibodies; CAR T, chimeric antigen receptor T‐cell therapy; CD19, cluster of differentiation 19; CD20, cluster of differentiation 20; EMA, European Medicines Agency; FDA, Food and Drug Administration.

These regulatory differences have major implications for real‐world access (Table 3). In the US and Canada, immediate post‐approval access is common, facilitated by expedited programs. However, in Europe, access is frequently delayed by a median of 7.5 months (range: 1–22 months) due to national reimbursement procedures, center qualification requirements, and the implementation of risk management plans [26, 29]. While the US benefits from quicker access, it faces challenges including high manufacturing costs, reimbursement issues, and access disparities by race and geography [25, 28]. Japan follows a different model, approving both CAR T and bispecific products through standard (non‐expedited) pathways, though detailed timelines are less frequently reported [26]. The contrasting regulatory environments highlight the need for harmonized frameworks to ensure timely and equitable patient access to innovative lymphoma therapies.

**TABLE 3:** Real‐world access challenges and timelines for CAR T‐cell therapies and BsAbs in rel/ref DLBCL by region.

Region	Time to access	Access requirements	Key implementation challenges
US	Immediate post‐FDA approval	Confirmatory trials, specialized centers	Cost, manufacturing, demographic disparities [25, 28]
Europe	Median 7.5 months (1–22 months)	National reimbursement, risk plans, certification	Reimbursement lag, infrastructure, referrals [26, 29]
Canada	Immediate (conditional)	Ongoing trials, conditional approval	Not clearly reported [27]
Japan	Regular approval	Not specified	Not reported [26]

Abbreviations: FDA, Food and Drug Administration; US, United States.

## 4 Clinical Efficacy of CAR T‐Cell Therapies Versus BsAbs: Trial‐Based Comparisons

CAR T Cell therapies and BsAbs have both demonstrated clinical benefit in rel/ref DLBCL, yet their efficacy profiles, safety outcomes, and trial frameworks reveal important distinctions [31, 32, 33, 34, 35, 36, 37, 38, 39, 40] (Table 4). Pivotal CAR T trials such as ZUMA‐1 (axicabtagene ciloleucel), JULIET (tisagenlecleucel), and TRANSCEND (lisocabtagene maraleucel) reported overall response rates (ORR) ranging from 52% to 80%, and complete response (CR) rates from 40% to 60% [31, 35, 41]. In ZUMA‐1, axi‐cel achieved an ORR of 74.3% and a CR rate of 54.5% [38], while JULIET reported an ORR of 52% and CR of 40% [31]. TRANSCEND reported up to 72.7% ORR with lisocabtagene maraleucel [35]. By comparison, bispecific antibodies such as epcoritamab (evaluated in EPCORE NHL‐1) and glofitamab (NP30179) have demonstrated ORRs between 52% and 73.4%, and CR rates between 36% and 48.5% [32, 34, 36, 38]. Glofitamab reported an ORR of 52% and CR of 39% [34], while epcoritamab reached 73.4% ORR and 48.5% CR in indirect comparisons with axi‐cel [38].

**TABLE 4:** Clinical efficacy comparison—CAR T vs. BsAbs in rel/ref DLBCL.

Therapy type	Therapy name(s)	ORR (%)	CR (%)	Median PFS (months)	1‐year OS (%)
CAR T	Axicabtagene ciloleucel	64–80 [31, 34, 41]	53–60 [31, 34, 41]	Up to 12.5 [37]	63.5 [36]
	Tisagenlecleucel	52–66 [31, 33]	40–42 [31, 33]	~11 [38]	48.8 [36]
	Lisocabtagene maraleucel	72.7 [35]	Not reported	Not reported	Not reported
BsAbs	Epcoritamab	52–73.4 [32, 34, 36]	36–48.5 [32, 34]	4.4 [37]	~50 [36]
	Glofitamab	52 [34]	39 [34]	4.4–12.6 [34]	50 [34]

Abbreviations: BsAbs, bispecific antibodies; CAR T, chimeric antigen receptor T‐cell therapy; CR, complete response; ORR, overall response rate; OS, overall survival; PFS, progression‐free survival; rel/ref DLBCL, relapsed/refractory diffuse large B‐cell lymphoma.

Recent phase III trials have moved CAR T‐cell therapy into the second‐line setting. The ZUMA‐7 trial (*axicabtagene ciloleucel*) and TRANSFORM trial (*lisocabtagene maraleucel*) showed better event‐free survival compared with standard salvage chemotherapy followed by autologous stem‐cell transplantation, leading to regulatory approval for second line use in large B‐cell lymphoma. This shift often places CAR T therapy before BsAbs in treatment sequencing. Pivotal BsAb trials, including EPCORE NHL‐1 (*epcoritamab*) and NP30179 (*glofitamab*), mainly enrolled heavily pretreated patients. Subgroup analyses suggest BsAbs can still be effective in patients previously treated with CAR T, and ongoing studies are examining their role after CAR T failure or as consolidation therapy [33, 39, 40, 41].

Progression‐free survival (PFS) and overall survival (OS) further differentiate the two classes. Axicabtagene ciloleucel demonstrated a median PFS of 12.5 months compared to 4.4 months with epcoritamab in one matching‐adjusted indirect comparison [37]. In the DESCAR T registry, 1‐year PFS was 46.6% for axi‐cel and 33.2% for tisa‐cel, with corresponding 1‐year OS rates of 63.5% and 48.8%, respectively [36]. Glofitamab showed a median PFS of 12.6 months, though this included a wider confidence interval and shorter follow‐up [34]. Meta‐analysis data also showed that 1‐year OS rates for BsAbs ranged from 32% to 50%, typically lower than those for CAR T products [32].

Safety profiles offer another area of differentiation (Table 5). CAR T therapies have higher toxicity burdens, particularly grade ≥ 3 CRS and neurotoxicity. ZUMA‐1 reported grade ≥ 3 CRS in 22% of patients and neurotoxicity in 12% [31, 42]. A pooled meta‐analysis found grade ≥ 3 CRS occurred in 8% (range 3%–12%) of CAR T recipients, with neurotoxicity in 11% (range 6%–17%) [32]. In contrast, BsAbs demonstrated lower grade ≥ 3 CRS (2%–4%) and neurotoxicity (1%–3%) [32, 34, 42]. For example, in the NP30179 glofitamab trial, only 4% experienced grade ≥ 3 CRS and 3% had grade ≥ 3 neurotoxicity [34]. Rates of grade ≥ 3 infections were 15%–20% in CAR T cohorts and 10%–15% in BsAbs trials [34, 42]. One study reported a 9% treatment discontinuation rate and 5% fatal serious adverse event rate for glofitamab [34].

**TABLE 5:** Grade ≥ 3 adverse events—CAR T vs. BsAbs.

Event type	CAR T (axicabtagene, tisagenlecleucel)	BsAbs (epcoritamab, glofitamab)
CRS ≥ Grade 3	4%–22% [31, 34, 42]	2%–8% [32, 34, 42]
Neurotoxicity ≥ Grade 3	3%–12% [31, 34, 42]	1%–3% [10, 34, 42]
Infections ≥ Grade 3	15%–20% [34, 42]	10%–15% [34, 42]
Serious adverse events	Up to 5% fatal [34]	Not consistently reported

Abbreviations: BsAbs, bispecific antibodies; CAR T, chimeric antigen receptor T‐cell therapy; CRS, cytokine release syndrome; Grade ≥ 3, Grade 3 or higher severity adverse events.

Cross‐trial comparisons remain limited by substantial heterogeneity. Several studies relied on matching‐adjusted indirect comparisons (MAICs) to evaluate CAR T versus BsAbs, especially axi‐cel versus epcoritamab [37, 38]. However, differences in trial design, eligibility, and patient characteristics remain major limitations. For instance, ZUMA‐1 excluded bridging therapy entirely, while JULIET allowed it in 90% of patients [31, 43]. Additionally, the median age of patients in the EPCORE NHL‐1 trial was higher (69.5 years), and real‐world CAR T populations often include more heavily pretreated or high‐risk individuals [34, 36]. Variability in follow‐up time may also underestimate relapse in bispecific trials. Therefore, although CAR T therapies appear to offer greater efficacy and durability of response, their toxicity and logistical complexity may favor the outpatient convenience and lower toxicity of BsAbs in selected patients.

## 5 Onset and Duration of Response

### 5.1 Temporal Response Kinetics and Durability Paradigms in Advanced Immunotherapies for B‐Cell Lymphomas

CAR‐T cell therapies, along with BsAbs, are emerging as a paradigm shift in the therapeutic approach for rel/ref DLBCL. Although both have utilized the T‐cell cytotoxic approach in CD19+ malignancies, the differing underlying mechanisms have provided essential points of difference concerning response kinetic elements, as the subject has been inadequately explored in current literature. In the lack of randomized trials for rel/ref DLBCL, essential lessons can be appropriately gathered from experiences in related lymphoproliferative malignancies, especially relapsed/refractory follicular lymphoma (RR‐FL) [43, 44, 45].

The phase II ZUMA‐5 study of axicabtagene ciloleucel (axi‐cel), a CAR T‐cell product, in RR‐FLT showed an unparalleled response rate of 94% with 79% CRs, along with a median duration of response of 38.6 months. What is most remarkable, however, is the demonstration of a median duration of response of > 60 months, along with a plateaued progression‐free survival of 50% at 5 years, setting a novel therapeutic standard for sustaining comprehensive disease control following a singular therapeutic procedure [43]. By contrast, the phase 2 Go29781 Clinical Trial of mosunetuzumab, a BsAb, showed a response rate of 78% with 60% CRs, as well as a median progression‐free survival of 24 months, implying strong, albeit temporary, efficacy mandating indefinite therapeutic continuity [45]. The contrast between these two therapeutic modalities aptly illustrates a paramount therapeutic truism, namely, while CAR T cells hold the promise of cure despite cumbersome manufacturability challenges as well as acute toxicities, BsAbs provide a rapid, safe, but nonetheless incessant, response.

On extrapolating these findings to DLBCL, it becomes absolutely essential to acknowledge the high degree of biologic heterogeneity as a responder to therapeutic action as a determining factor. In a pioneering study, metabolic architectural heterogeneity as quantitatively captured using advanced ^18^F‐Fluordeoxyglucose Positron Emission Tomography/Computed Tomography (^18^F‐FDG PET/CT)‐radiomics has been recognized as a vital determining factor deciding both response rates as well as velocity. In a first‐of‐its‐kind study evaluating 180 DLBCL‐treated patients undergoing CAR‐T therapy, higher pre‐therapeutic MTV as well as higher SUVmax were identified as independent predictors for decreased complete response rates, shorter progression‐free survival, as well as higher CRS severity [46]. More advanced mathematical modeling has also enabled the development of high‐dimensional radiomics features, which combined lesion morphology, heterogeneity, as well as metabolic maps, significantly outperforming simple volumetric as well as intensity measures as predictors of therapeutic response, yielding higher AUC values of 0.73 compared to 0.66, 0.59, respectively [47].

Paradigm‐shifting findings have confirmed the efficacy of early PET scans following treatment at day 30 post‐infusion in stratifying patients according to Deauville criteria, where a score of 1–2 signifies a state of prolonged remission, while a score of 5 signifies expected therapeutic failure definitively [43]. Elaborate spatial–temporal correlation mapping has unraveled the fact that tumors encompassing complex irregular morphologies along with high metabolic activities display expedited immune escape pathways along with transient persistence of CAR‐T cells [47]. It has been demonstrated, consequently, that BsAbs like glofitamab induce initial complete responses within approximately six weeks, while durability varies significantly based on analogous biological properties of tumors [48].

These convergent results trigger a paradigm‐shifting transformation, whereby the complexity of tumor microenvironmental morphology and metabolic profile, rather than the choice of immunotherapy platform per se, radically dictates response pharmacodynamics. Conceptually, this approach debunks simplistic reductionism concerning the choice of therapeutic, mandating the incorporation of novel imaging biomarkers, radiomics, and molecular characterization into therapeutic algorithms [49, 50]. Thus, a precision medicine strategy would facilitate individualized therapeutic sequencing, potentially deferring the use of BsAbs for those warranting rapid control of disease progression with reduced acute toxicity, while prioritizing CAR T‐cell therapy for those warranting a pre‐defined biological signature of long‐term immunologic memory. The putative implementation of this biology‐informed therapeutic approach promises a potentially transformative resetting of the efficacy‐toxicity curve for aggressive B‐cell lymphoma patients.

## 6 Safety and Adverse Events

### 6.1 CAR T‐ Cell Therapy: Safety and Adverse Events

Though CAR T‐cell therapy has enhanced outcomes for patients with DLBCL who have relapsed or refractory, it has been associated with severe and sometimes unexpected toxicities. The incidence of CRS ranges from 42% to 93%, with 8% (range: 3%–12%) experiencing severe (grade ≥ 3) CRS. Within days of infusion, CRS typically manifests as fever, hypotension, and hypoxia [47]. ICANS affects 14%–40% of patients; approximately 11% (range: 6%–17%) experience severe (grade ≥ 3) events. Symptoms include confusion, aphasia, convulsions, and rarely, cerebral oedema. Most neurotoxicities are reversible, but close monitoring is essential [51, 52]. Prolonged cytopenias may increase the risk for bleeding and infection. Thrombocytopenia, anemia, and neutropenia are common, especially in the first six months post‐infusion [47, 48]. Long‐term follow‐up demonstrated that infections occurred at a rate of 5.6 per 100 person‐months and thus were a significant late complication. Severe infections (grade ≥ 3) occurred in 11%–22% of all patients and are a leading cause of non‐relapse mortality [51, 52].

The safety profiles of these treatments have been revealed by the important ZUMA‐1 (axi‐cel), JULIET (tisa‐cel), and TRANSCEND (liso‐cel) trials. The primary safety findings from these studies are displayed in Table 6. It demonstrates how toxicities such as CRS, ICANS, cytopenias, and infections varied in frequency and how they were treated [54].

**TABLE 6:** Key safety outcomes from ZUMA‐1, JULIET, and TRANSCEND trials.

Safety outcome	ZUMA‐1 (axi‐cel) [5]	JULIET (tisa‐cel) [10]	TRANSCEND (liso‐cel) [53]
CRS, any grade	92%	58%	42%
CRS, grade ≥ 3	10%	22%	2%
Neurotoxicity (ICANS), any grade	67%	21%	30%
Neurotoxicity, grade ≥ 3	32%	11%	10%
Median onset of CRS (days)	2	3	5
Median resolution of CRS (days)	8	7	5
Median onset of ICANS (days)	5	6	9
Median resolution of ICANS (days)	17	14	11
Tocilizumab use (%)	43%	14%	20%
Corticosteroid use (%)	27%	10%	21%
ICU admission due to toxicity (%)	Not reported	24%	4%
Prolonged cytopenias (≥ 28 days, grade ≥ 3)	38%	32%	37%
Neutropenia ≥ 28 days	26%	24%	60%
Thrombocytopenia ≥ 28 days	24%	41%	27%
Anemia ≥ 28 days	10%	Not reported	37%
Infections (grade ≥ 3)	28%	20%	12%
Hypogammaglobulinemia (grade ≥ 3)	0%	Not reported	0%
Treatment‐related mortality	2%	0%	1%

*Note:* Data reflect patients from published trials, with event timing stratified by ≤ 8 weeks and > 8 weeks post‐infusion.

Abbreviations: CRS, cytokine release syndrome; ICANS, immune effector cell‐associated neurotoxicity syndrome.

The safety profiles of CAR T cells directed against CD19 for the treatment of rel/ref DLBCL differ significantly between products. In the core studies, CRS and ICANS were the most frequent toxicities, even though with different incidences and severities [54]. Axi‐cel (ZUMA‐1) showed the highest incidence of CRS (92%) and ICANS (67%), with grade ≥ 3 episodes happening in 10% and 32% of patients, respectively [5]. Compared to tisa‐cel, which exhibited intermediate toxicity [51], liso‐cel had a more favorable safety profile, characterized by significantly lower rates of grade ≥ 3 CRS (2%) and ICANS (10%) [53].

The supportive care interventions followed the toxicity profiles; tocilizumab administration and corticosteroid administration were highest in ZUMA‐1, 43% versus 14% in JULIET, 20% in TRANSCEND; 27% versus 10% in JULIET, 21% in TRANSCEND. Admissions to the intensive care unit were higher in JULIET, 24% versus 4% in TRANSCEND; ZUMA‐1 did not record this endpoint. Long‐term cytopenias were reported in all studies, grade ≥ 3 neutropenia in 60% of the participants in TRANSCEND. Infection rates, grade ≥ 3, were highest in ZUMA‐1, 28% versus 12% in TRANSCEND. The results point towards all three CAR T products being able to achieve a long‐lasting response; however, the toxicities can be entirely different. Even though the main safety findings from the JULIET, TRANSCEND, and ZUMA‐1 studies are presented together, they ought not to be compared. The difference in research design, distribution, distribution of grade, administration of supportive care, as well as eligible participants, can affect final findings. The safety of these CAR T cell products has not been definitively evaluated in randomized head‐to‐head comparison studies [54].

### 6.2 BsAbs: Safety and Adverse Event

BsAbs mark a substantial advancement in the management of rel/ref large B‐cell lymphoma (LBCL), especially in diffuse large B‐cell lymphoma (DLBCL). In spite of their potential benefits, these therapeutic agents have been observed to have diverse toxicities, mainly attributed to T‐cell activation and B‐cell exhaustion. The following is a comprehensive outline of substantial adverse effects, as per available clinical evidences. Adverse effects associated with BsAbs, such as epcoritamab, glofitamab, mosunetuzumab, and odronextamab, have a common safety profile, as indicated in numerous studies conducted on these therapeutic agents [55]. The efficacy and safety of nine BsAbs were recently examined in a comprehensive systematic study conducted on 19 interventional clinical studies from 2016 to 2024. The study considered the BsAbs as standalone therapy as well as combinations, which specifically target CD19/CD3 or CD20/CD3. Focusing on CRS, ICANS, infections, cytopenia, fatigue, as well as other toxicities, the comprehensive study highlighted diverse Adverse Reaction Events associated with these therapeutic agents [56].

Table 7 illustrates the occurrence of adverse events in LBCL patients treated with BsAbs. The immune‐related adverse event, CRS, was the most frequently reported, though it has a notable variability among 19 studies, ranging from 0% to 72.2% for any grade CRS. For grade ≥ 3 CRS, it has been lower, ranging from 1.3% to 7% among nine studies. Both combinations of regimens and targets of BsAbs did not affect the CRS occurrence, while all studies had a management strategy consisting of premedications, step‐up infusions, as well as outpatient care, to decrease CRS frequency [56]. ICANS has been observed in 15 studies, though infrequent. The highest frequency of ICANS, 27%, has been observed in the CD19/CD3 BsAb AZD0486 study, a first‐in‐human study, whose step‐up infusion approach has not been fully evolved, while a CD20/CD3 BsAb has a median ICANS from 0% to 12% in eight studies. In a CD19/CD47 BsAb, TG‐1801, without CD3 T cells, ICANS has not been observed, so it does not use CD3 T cells for execution [56]. Infection has been a remarkable treatment‐emergent adverse event, reported in eight studies. The grade ≥ 3 infections have a variability ranging from 14.6% to 23% while from 41.6% to 49% for any grade, where the highest frequency has been observed in 13% for patients having severe infections in the form of pneumonia. In the 12 studies, anti‐infective prophylactic medication has been injected following the institutional criteria. Fever has a variable frequency. The highest frequency has been observed in 18.2% to 73% for any grade fever, among 11 studies, while grade ≥ 3 fever has been observed in only three studies. Fatigue, another non‐hematologic toxicity, has been highest, observed in 14 studies, ranging from 17.9% to 46.7%, especially while combining mosunetuzumab [56].

**TABLE 7:** Adverse events associated with BsAbs in LBCL patients across major clinical trials [5, 10, 54].

Bispecific antibody	CRS (any grade)	CRS (≥ grade 3)	ICANS (any grade)	ICANS (≥ grade 3)	Infections (any grade)	Infections (≥ grade 3)	Fever (any grade)	Fever (≥ grade 3)	Fatigue (any grade)	Fatigue (≥ grade 3)
Blinatumomab	0	0	Not reported	Not reported	Not reported	Not reported	43.5%	4.3%	26.1%	0%
AZD0486	48%	0	27%	9.7%	Not reported	Not reported	Not reported	Not reported	Not reported	Not reported
TG‐1801	0	0	0%	0%	Not reported	Not reported	Not reported	Not reported	25%	0%
Odronextamab	61%	7%	12%	3%	49%	23%	73%	1%	33%	5%
Glofitamab	50.2%	2.6%	4%	1.5%	49%	17.5%	18.2%	0%	17.9%	0.6%
Epcoritamab	46.4%	2.3%	2.5%	0%	41.6%	14.6%	31.8%	0%	25%	1.9%
Plamotamab	72.2%	0%	Not reported	Not reported	38.9%	Not reported	Not reported	Not reported	Not reported	Not reported
GB261	12.8%	0%	0%	0%	NR	Not reported	Not reported	Not reported	Not reported	Not reported
Mosunetuzumab	39.4%	2.5%	4.9%	1.6%	46.8%	15.1%	20%	0.9%	46.7%	6.7%

*Note:* CRS and ICANS rates are based on published trial data from nine BsAb agents targeting CD19/CD3 or CD20/CD3 in LBCL patients.

Abbreviations: BsAb, bispecific antibody; CRS, cytokine release syndrome; ICANS, immune effector cell–associated neurotoxicity syndrome; LBCL, large B‐cell lymphoma.

All 19 studies noted cytopenias, primarily anemia, thrombocytopenia, and neutropenia. Anemia grade ≥ 3 rates were up to 25% and any grade from 4.4% to 44.5%; thrombocytopenia rates from 11.5% to 40.7% for any grade and 5.7% to 17.4% grade ≥ 3; neutropenia grade ≥ 3 rates from 14.6% to 25% and any grade from 4.4% to 36.5%, highest at 70% in the mosunetuzumab + CHOP combining study, which also reported highest total cytopenia incidence. Leukopenia, a form of cytopenia, as well as lymphopenia, were observed in fewer studies [56]. Other SAEs included neurological, gastrointestinal, as well as electrolyte disturbances. For example, a Blincyto study identified tremors at 47.8% incidence, grade ≥ 3 encephalopathy 8.7%, and aphasia 8.7% incidence. The Odronextamab monotherapy showed 47% incidence of chills, 29% any grade, and 19% grade ≥ 3, for SAEs of hypophosphatemia. No significant difference in SAE's proportion were observed between monotherapy versus combinations or BSAB's of CD 19 versus CD 20 antigens [55, 57].

The toxicity profiles of BsAbs are quite comparable to those reported in other hematologic malignancies. Despite the high incidence of CRS in BsAbs, it is rare to have serious CRS. In comparison to CAR T‐cell therapy, BsAbs had lower rates of ICANS (0%–27% vs. 21%–67%) and CRS (15%–80% vs. 42%–93%). The observed lower rates could be attributed to the step‐up approach and relatively reduced T‐cell activation compared to CAR T cells. The rates of cytopenias and serious infections were also lower in BsAbs compared to CAR T therapy. Nevertheless, repetitive administration of BsAbs, suggesting a continuous depletion of B cells, poses a potential question on long‐term toxicity, especially concerning hypogammaglobulinemia, which has been well documented in CAR T cells. Unlike traditional lymphoma therapies, including rituximab, cyclophosphamide, doxorubicin hydrochloride (hydroxydaunorubicin), vincristine sulfate (Oncovin), and prednisone (R‐CHOP), antibody‐drug conjugates, and conventional chemotherapy, BsAbs did not entail toxicities such as skin rashes, mucositis, and cardiac toxicity. The toxicities included fatigue, fever, and PN; the latter, though, could be relatively rare, potentially due to the lack of microtubule‐targeted drugs [56].

Table 8 provides a comparison of the AEs of CAR‐T therapy versus those of BsAbs. The rates of any grade CRS were 42% to 93% vs. 0% to 72.2% for CAR‐T vs. BsAb, while grade ≥ 3 CRS were 8% (3% to 12%) vs. 2% to 4% respectively. The rates of grade ≥ 3 neurotoxicity were 11% (6%–17%) vs. approximately 1% for CAR‐T vs. BsAb, respectively. The rates of cytopenias were up to 80% versus 70% for CAR‐T versus BsAb, respectively. The rates of grade ≥ 3 infections were 17% (11%–22%) versus 10% (3%–16%) for CAR‐T versus BsAb, respectively. The rates of treatment interruptions were not available for CAR‐T, but up to 53% for BsAb. The off‐the‐shelf readiness is a distinct advantage of BsAbs, as they can be directly administered as opposed to the weeks' long processing time required for CAR‐T cells administration [5, 56].

**TABLE 8:** Comparative toxicity profile of CAR T‐cell therapy and BsAbs in rel/ref DLBCL [10, 53].

Adverse event	CAR T‐cell therapy^a^	BsAbs^a^
CRS (any grade)	42%–93%	0%–72.2%
CRS (grade > or = 3)	8% (3%–12%)	2%–4%
Neurotoxicity (grade > or = 3)	11% (6%–17%)	1% (rare)
Cytopenias	Up to 80%	Up to 70%
Infections (grade > or = 3)	17% (11%–22%)	10% (3%–16%)
Treatment interruption	Not routinely reported	Up to 53%
Off‐the‐shelf availability	No	Yes
Manufacturing time	Weeks	Immediate

Abbreviations: CAR T‐cell, chimeric antigen receptor T‐cell therapy; CRS, cytokine release syndrome; R/R DLBCL, relapsed/refractory diffuse large B‐cell lymphoma.

^a^ Data derived from multiple phase 1/2 trials and systematic reviews. Percentages reflect incidence ranges across major studies.

## 7 Accessibility and Logistical Burden

The manufacturing processes of CAR T cells and BsAbs reveal sharp contrasts in accessibility and associated logistical burden. CAR T cell therapy depends on an elaborate, patient‐specific production cycle that often spans 3–4 weeks. These include leukapheresis, genetic engineering of autologous T cells to express CARs, and ex vivo expansion under GMP conditions before reinfusion [58, 59]. Such steps necessitate specialized infrastructure and highly trained personnel, often available only in certified tertiary centers located in urban or high‐income regions [58, 59]. Consequently, access to CAR T therapy remains geographically and socioeconomically limited. For instance, only one‐third of African Americans live in counties with access to CAR T clinical trials, and studies suggest that 29%–71% of eligible patients fail to receive approved CAR T products [58, 59].

In contrast, BsAbs are “off‐the‐shelf” agents that can be administered without the need for individualized manufacturing or apheresis, thus offering the possibility of rapid initiation of treatment, an important advantage in patients with aggressive disease or with limited access to specialized centers [58, 59]. BsAbs also diminish logistical barriers to health care systems since they can be delivered in a wider range of facilities without requirements for cell processing labs. However, BsAbs are administered via a chronic model of care, with agents such as glofitamab administered for a fixed duration up to 12 cycles and others such as epcoritamab continued until disease progression [60]. This leads to cumulative resources used over time; although this avoids the intensive inpatient monitoring that is often required for CAR T therapies, BsAbs administration does result in oncology clinic resource use [59, 60].

Worldwide, the gap in accessibility continues. Globally, CAR T therapies remain concentrated in high‐income countries, with limited penetration in lower‐income regions. Emerging programs in India and Latin America are trying to increase availability, but treatment cost, requirements for inpatient care, and proximity to certified infusion centers continue to disproportionately affect vulnerable populations [59, 60]. As such, scalability and feasibility give BsAbs as an accessible option in many health setups, especially in resource‐constrained settings [59].

## 8 Cost and Reimbursement

Another hugely influential cost factor is the choice between CAR T cell therapy and BsAbs. Estimated costs for CAR T therapy can reach upwards of $350,000, with particular therapies like axicabtagene ciloleucel initially priced at $373,000, now increasing to $424,000 in the US. By contrast, BsAbs are significantly less expensive, costing between $15,000–$30,000 per dose, though prices for more recently approved drugs, such as epcoritamab and glofitamab, have reached approximately $37,500 and $41,176 per month, respectively [58, 60]. Although the CAR T is considered cost‐effective compared to chemotherapy regimens and stem cell transplants, cost‐effectiveness studies comparing CAR T to BsAbs in the third‐line setting are needed [59]. A hypothetical cohort study found that axi‐cel could be cost‐effective at a WTP of $100,000/QALY, increasing to $271,399/QALY in third‐line settings, bringing up concerns about the cost‐effectiveness of CAR T at that stage [58]. While there are high costs associated with these treatments, the potential ability of outpatient administration of CAR T therapies to lower overall healthcare spending, especially those with a lower frequency of severe adverse events, such as liso‐cel, is of great interest [58]. However, the price of BsAbs such as blinatumomab at an estimated $89,000 per cycle calls the idea that BsAbs are cheaper than CAR T into question when considering a treatment‐until‐progression paradigm [58]. Reimbursement for these therapies is complex and involves navigating several key financial considerations. Patients rely on their health insurance to pay a large portion of costs, but actual coverage varies wildly. Reimbursement rates are negotiated by healthcare providers and pharmaceutical companies, often through outcomes‐based agreements that depend on the effectiveness of the treatments [60]. Patients with private insurance tend to experience longer delays in accessing CAR T therapies compared to patients with government insurance [60]. Implementation of CAR T therapies has focused mostly in high‐income geographies, with very limited access in Latin America due to both regulatory and systemic challenges [56]. Ongoing direct cost‐effectiveness comparisons and real‐world outcome studies are much needed and must be done in order to optimize patient access and inform treatment decisions.

## 9 Patient Eligibility and Real‐World Utilization

The inclusion and exclusion criteria of CAR T therapy and BsAbs remain some of the most significant challenges. CAR T trials have strict age, performance status, and renal function restrictions, which disproportionately exclude older adults and patients with comorbidities. Moreover, race‐specific barriers, such as benign ethnic neutropenia common in African Americans, result in disqualification for low absolute neutrophil count, despite this being a normal variant. In contrast, while trials for BsAbs, for example, anti‐CD20, also include hematologic exclusion criteria, they may adopt slightly broader comorbidity criteria; however, they still have the tendency to exclude high‐risk populations, especially those with rapid disease progression following CAR T [59, 60, 61]. This strict eligibility framework thus limits the enrolment of older, racially diverse, and comorbid patients. These factors skew trial results and diminish real‐world applicability, as shown by the underrepresentation of Black and Hispanic patients in CAR T trials, with only 1% Black enrolment in myeloma CAR T trials compared with 16.6% in non‐CAR T groups. More concerning is the dropout rates pre‐infusion, where up to 5.9% of Black B‐ALL patients do not receive CAR T because of disease progression or logistical barriers. Delays in referral and manufacturing processes further contribute to these dropout rates, with rural and low socioeconomic status patients being at an increased risk due to travel difficulties. These dropouts compromise trial generalizability and further widen the gap between the enrolled population and the real‐world population. Finally, expanded access programs for BsAbs aim to address these gaps for ineligible patients by providing earlier access; however, they also demonstrate logistical limitations, including site availability, as was demonstrated by the absence of open CAR T trials in 20 states [61].

## 10 Resistance Mechanisms and Sequential Use

CAR T cell therapy and BsAbs represent two leading immunotherapeutic options for rel/ref DLBCL, yet their failure patterns and resistance mechanisms are distinct and clinically relevant [58, 59, 60, 61, 62, 63] (Table 9). Resistance to CAR T therapy has been extensively documented and often involves CD19 antigen loss, either via deletion, epitope mutation, or lineage switch, as well as T‐cell exhaustion and the influence of an immunosuppressive tumor microenvironment [62, 66, 68]. CD19 loss was observed in 2 of 34 patients in institutional studies, 3 of 11 in ZUMA‐1, and 1 of 5 in JULIET [63]. Some studies report relapse rates post‐CAR T approaching 60% [64, 66, 68]. The presence of high‐risk features such as double‐hit lymphoma and poor IPI scores further correlate with inferior CR and PFS [55]. T‐cell dysfunction, tumor trafficking deficits, and microenvironmental suppression contribute to relapse, and despite the use of next‐generation CAR constructs or dual‐targeting strategies (e.g., CD19/CD20), resistance often results in rapid clinical progression [63, 64, 66].

**TABLE 9:** Resistance mechanisms in CAR T therapy vs. BsAbs.

Category	CAR T therapy	BsAbs
Primary antigen target(s)	CD19	CD20 (glofitamab), CD3/CD20 (epcoritamab), CD22 (experimental) [64]
Main resistance mechanism(s)	CD19 antigen loss (deletion, mutation, lineage switch), epitope loss, T‐cell exhaustion, suppressive tumor microenvironment [62, 63, 65, 66, 67, 68, 69, 70]	T‐cell dysfunction, LBCL‐intrinsic tumor biology, immune escape [62, 66, 68]
Reported frequency	CD19 loss: 2/34 (Bukhari), 3/11 (ZUMA‐1), 1/5 (JULIET); relapse rate post‐CAR T: up to 60% [65, 67, 68, 69]	Quantitative data not reported; theoretical resistance inferred from low responses [66, 68]
Impact on clinical outcomes	Poor CR/PFS, early relapse, dismal survival, limited response to subsequent therapy [62, 64, 66, 68, 69]	ORR 43%, CR ~35%, PFS 2.8 months in post‐CAR T setting (Shumilov) [71]
Prognostic factors linked to resistance	High IPI score, double‐hit lymphoma, high LDH [62, 67, 68]	LBCL‐intrinsic features, elevated LDH [71]
Potential interventions	Dual‐target CAR T (e.g., CD19/CD20), next‐generation constructs, early sequencing [65, 68]	Currently limited; theoretical benefit from dual‐target CAR T after bispecific failure [65, 68]

Abbreviations: BsAbs, bispecific antibodies; CAR T, chimeric antigen receptor T‐cell therapy; CR, complete response; IPI, international prognostic index; LBCL, large B‐cell lymphoma; LDH, lactate dehydrogenase; ORR, overall response rate; PFS, progression‐free survival.

In contrast, resistance to BsAbs, though less well characterized, appears linked to T‐cell dysfunction, tumor immune escape, and overlapping mechanisms with CAR T resistance such as Large B cell Lymphoma (LBCL)‐intrinsic biology [62, 64, 65, 69]. While CD19 antigen escape is mitigated in bispecifics that target CD20 or CD22, the absence of quantitative data hampers definitive conclusions. Only a few studies report clinical resistance to bispecifics post‐CAR T, with one retrospective cohort showing an ORR of 43%, a CR rate of approximately 35%, and a median PFS of 2.8 months following CAR T failure [64]. These outcomes suggest modest benefit and highlight the limited durability of sequential therapy.

When CAR T is followed by BsAbs treatment, the evidence shows feasibility but limited efficacy (Table 10). Shumilov et al. reported an ORR of 43% and CR of ~35% in post‐CAR T patients receiving BsAbs, with PFS again at 2.8 months [64]. Similar figures were echoed in narrative reviews and meta‐analyses [62, 65]. Conversely, data on using CAR T after bispecific failure are virtually absent, with no robust trials directly addressing this sequence. One phase I study evaluating bispecific CAR T targeting both CD19 and CD20 demonstrated promising results (ORR 82%, CR 55%) and no antigen loss, although the study was not limited to DLBCL and remains investigational [60]. Furthermore, several studies emphasize the need for novel sequential protocols, especially for patients who relapse early post‐CAR T, as they tend to show poor responses to bispecific salvage and carry dismal prognosis [64, 67].

**TABLE 10:** Clinical outcomes following treatment failure after CAR T‐cell and BsAbs therapies in rel/ref DLBCL.

Treatment sequence	Overall response rate (ORR)	Complete response (CR)	Progression‐free survival (PFS)	Key notes
CAR T → BsAbs	43% [68]	~35% [65]	2.8 months [68]	Limited efficacy; worse in early relapse, high LDH, poor‐risk disease [66, 68]
CAR T → Dual‐target CAR T	82% [62]	55% [59]	Not reported	Only in phase I, not specific to DLBCL; no antigen loss observed [62]
BsAbs → CAR T	Not reported	Not reported	Not reported	No published outcome data; lack of evidence for reverse sequencing [68, 69]
CAR T → Relapse (no further Tx)	~60% relapse [66, 68]	Not reported	Poor survival	Multifactorial resistance; poor prognosis without salvage options [64, 67, 69]

Abbreviations: BsAbs, bispecific antibodies; CAR T, chimeric antigen receptor T‐cell therapy; CR, complete response; DLBCL, diffuse large B‐cell lymphoma; LDH, lactate dehydrogenase; ORR, overall response rate; PFS, progression‐free survival; Tx, treatment.

Overall, CAR T therapy remains superior in terms of depth and durability of response, but its vulnerability to immune escape via CD19 loss and complex resistance biology underpins the rationale for BsAbs as a salvage option (Table 11). Despite their lower toxicity profile and outpatient feasibility, bispecifics yield limited outcomes when used after CAR T failure. Future strategies will likely involve dual‐targeting constructs, early identification of resistance mechanisms, and rational combination/sequential approaches, though current evidence underscores that such sequencing offers only incremental benefit and is highly dependent on disease biology and timing of relapse.

**TABLE 11:** Summary of sequential and combined therapy approaches.

Strategy	Study reference(s)	Population	Key findings
CAR T → BsAbs	Shumilov et al. [64], Melody and Gordon [65]	rel/ref DLBCL post‐CAR T	ORR ~43%, CR ~35%, PFS 2.8 months; feasible but limited outcomes
BsAbs → CAR T	Not reported	Not reported	No outcome data or case series identified
Dual‐target BsAbs CAR T	Bukhari et al. [63]	Early‐phase, not DLBCL‐specific	ORR 82%, CR 55%, antigen loss prevented; experimental phase I
Combined or concurrent use	Not reported	Not reported	No clinical data; theoretical interest only

Abbreviations: BsAb, bispecific antibody; CAR T, chimeric antigen receptor T‐cell therapy; CR, complete response; ORR, overall response rate; PFS, progression‐free survival; R/R DLBCL, relapsed/refractory diffuse large B‐cell lymphoma.

## 11 Combination Strategies and Future Directions in rel/ref DLBC

Combination strategies and pipeline innovations are driving the next wave of transformative immunotherapies within the evolving therapeutic landscape for rel/ref DLBCL. As impressive as the results have been, CAR T‐cell therapies and BsAbs also present challenges such as relapse, antigen escape, and treatment‐associated toxicities, hence a need for enhanced regimens. New emerging approaches now focus on synergistic combinations that augment T‐cell activation, overcome immune suppression, and improve durability of response [69, 70, 71, 72].

One of the most promising strategies includes combination approaches with BsAbs, such as epcoritamab or glofitamab, along with immunomodulatory agents like lenalidomide, which enhance T‐cell proliferation and cytotoxic potential and reshape the tumor microenvironment [69]. Furthermore, combinations of BsAbs with checkpoint inhibitors (such as anti‐programmed cell death protein 1 or anti‐programmed death‐ligand 1 antibodies) are capable of restoring T‐cell effector functions due to a reversal of tumor‐induced exhaustion, leading to enhanced tumor clearance [70]. This effect is strongly pronounced in tumors when malignant B cells express PD‐L1, which normally exerts its immune checkpoint function to dampen T‐cell responses.

Innovative protein engineering has also led to the development of dual‐targeting BsAbs with the capability of binding to more than one B‐cell antigen, such as CD19/20 or CD19/22. These constructs aim at reducing the risk of antigen escape—a known resistance mechanism in single‐target therapies—while increasing the breadth and depth of tumor recognition [72]. Preclinical and early clinical data suggest that this strategy may significantly improve response rates while lowering relapse risk. Parallel to this, CAR T‐cell platforms continue to evolve with the development of “armored” CARs or otherwise named TRUCKs—T cells Redirected for Universal Cytokine‐mediated Killing. These next‐generation constructs carry inducible cytokine modules, mainly IL‐12, which is selectively released in the tumor microenvironment following antigen engagement [71]. Localized IL‐12 delivery potentiates T‐cell cytotoxicity, recruits innate immune effectors, and reverses immunosuppressive signaling within the tumor niche without eliciting systemic toxicity commonly associated with recombinant cytokine therapy [71].

Finally, the integration of precision oncology tools such as genomic profiling, circulating tumor DNA (ctDNA) analysis, and radiomics is shaping a paradigm shift towards personalized immunotherapy. By tailoring treatments against individual tumor genotypes, immune phenotypes, and dynamic biomarkers, clinicians are able to optimize therapeutic outcomes while minimizing adverse events [73]. Liquid biopsy platforms and real‐time immune monitoring are particularly useful in guiding therapy selection and tracking disease evolution in patients undergoing CAR T‐cell or BsAbs treatment [50, 73].

## 12 Quality of Life and Patients' Preference

While clinical efficacy and survival remain central in relapsed/refractory DLBCL, patient‐centered outcomes such as QoL, treatment convenience, and individual preferences are increasingly important as the possibility of long‐term remission with newer immunotherapies becomes a reality. Guidelines now recommend shared decision‐making between CAR T therapy and BsAbs that incorporates these factors [66].

Car T therapy can be potentially curative, with durable complete responses in 40%–54% of patients in the pivotal trials including ZUMA‐1 and JULIET [70, 72]. However, it requires apheresis, lymphodepleting chemotherapy, and inpatient monitoring for CRS and neurotoxicity, occurring in 42%–93% and 12%–62% of patients, respectively [71]. Manufacturing delays and geographic limitations to proximity to certified centers limit access, adversely affecting patients, often elderly or frail [74].

BsAbs like epcoritamab and glofitamab offer an off‐the‐shelf outpatient option with favorable safety. Severe CRS occurs in less than 5% of patients, and neurotoxicity is rare [73]. Step‐up dosing schedules minimize hospital time and reduce disruption to work and daily life, which is a major factor in patient preference [75]. QoL as assessed by FACT‐Lym and EORTC QLQ‐C30 demonstrates that BsAb recipients report less fatigue, better sleep, and lower psychological distress compared with CAR T recipients. In a recent real‐world cohort, 78% of BsAb patients maintained or improved QoL over three months compared with 41% of CAR T recipients [73, 74, 76].

Treatment choice is influenced by patient values. Younger, fit patients may accept the intensive CAR T pathway for potential cure, whereas older patients or those who value convenience and rapid symptom relief often prefer BsAbs [75].

## 13 Challenges and Global Disparities in Implementing Advanced Immunotherapies

Still, the global accessibility for these immunotherapies in rel/ref DLBCL is unequal. While high‐income countries can offer CAR T and BsAbs, availability is limited in LMICs [10, 76, 77, 78, 79, 80, 81, 82, 83, 84, 85]. It is a highly effective but expensive and complex modality, with specialized infrastructure required for toxicity management related to CRS and ICANS [78]. Logistical and financial barriers, including systemic barriers, are accentuated in real‐world data, with treatment exceeding US$ > 350,000 in the US [86]; disparities have also been described within wealthy nations [79, 87].

BsAbs, such as glofitamab, exhibit high response rates and can be administered outpatient, reducing infrastructure demands [77, 80, 88, 89]. Costs, ranging from $15,000 to $30,000 per dose, along with limited LMIC trial representation, remain key barriers to access [68, 81, 86, 87]. Patient‐centered outcomes as measured by tools like FACT‐Lym reveal an improved quality of life for BsAbs and differential impacts compared with CAR T [82, 83, 84, 85].

Such initiatives as WHO and CARAMA support decentralized CAR T manufacturing, workforce training, biosimilar development, tiered pricing, and regulatory harmonization. Integration of the views of patients in decision‐making is stressed. Closing global gaps will necessitate sustained investment, coordinated policy, and international collaboration to ensure these therapies reach beyond a privileged few [86, 87, 88].

## 14 Conclusion

CAR T‐cell therapy and BsAbs have transformed the therapeutic landscape of rel/ref DLBCL with complementary immunotherapeutic options that carry different strengths and weaknesses. Where CAR T‐cell therapy has given durable remissions to selected patients, this modality remains restricted by high costs, logistical complexity, and toxicity. On the other hand, BsAbs offer immediate, scalable, and better‐tolerated alternatives, although with less‐proven long‐term durability. Optimizing their use requires a precise selection of patients and equity in access. Together, these innovations mark a significant step forward towards personalized and effective care for DLBCL worldwide.

## 15 Recommendations and Future Perspectives

Future efforts should be directed at the identification of predictive biomarkers and refinement of patient selection to optimize outcomes of CAR T‐cell therapy and BsAbs. Novel strategies, such as combination regimens and next‐generation constructs, are required to overcome resistance and improve durability of response. Policy reform, equitable trial enrollment, and scalable manufacturing must be used to address global disparities in order to expand access. Lastly, approaches that incorporate quality‐of‐life measures and patient preferences into treatment decisions will serve to better align innovations with patient needs.

## Author Contributions

All authors contributed equally to the conceptualization, drafting, and revision of the manuscript. All authors reviewed and approved the final version of the manuscript.

## Funding

The authors have nothing to report.

## Ethics Statement

The authors have nothing to report.

## Consent

The authors have nothing to report.

## Conflicts of Interest

The authors declare no conflicts of interest.

## Data Availability

Data sharing not applicable to this article as no datasets were generated or analyzed during the current study.
